# Two new species of the genus *Deltomerodes* Deuve, 1992 (Coleoptera, Carabidae, Patrobini) from Xizang, China

**DOI:** 10.3897/zookeys.1023.61553

**Published:** 2021-03-10

**Authors:** Weifeng Yan, Hongliang Shi, Hongbin Liang, Juan Shi

**Affiliations:** 1 College of Forestry, Beijing Forestry University, Beijing 100083, China Beijing Forestry University Beijing China; 2 Key Laboratory of Zoological Systematics and Evolution, Institute of Zoology, Chinese Academy of Sciences, Beijing 100101, China Institute of Zoology, Chinese Academy of Sciences Beijing China

**Keywords:** Alpine insect, key, morphology, Tibetan Plateau

## Abstract

Two new species of the carabid genus *Deltomerodes* are described from Xizang (China): *D. ovicollis***sp. nov.** (type locality: Doxong La, Mêdog) and *D. conaensis***sp. nov.** (type locality: Rama La, Cona). The new species are amply described, illustrated, and their distributions are mapped. An updated key to all eight known Chinese *Deltomerodes* species is provided.

## Introduction

**Map 1.**
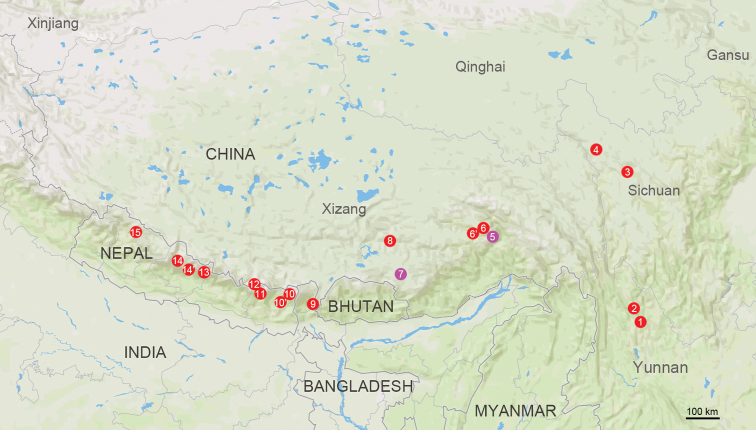
Distribution map for *Deltomerodes* species. Distributions are listed in the checklist below (magenta for new species, red for recorded species).

*Deltomerodes* Deuve, 1992 (Patrobini, Deltomerodina), the sole genus of the subtribe Deltomerodina Zamotajlov, 2002, is a small genus, previously containing thirteen species (Map 1), known from China, Nepal and India (Zamotajlov 2017). This subtribe was erected for the most primitive monophyletic group of the tribe Patrobini Kirby, 1837, the sister group to the other three subtribes based on the results of a phylogenetic analysis (Zamotajlov 2002). In China, six species of *Deltomerodes* have been recorded so far in Yunnan, Sichuan and Xizang (Deuve 1992; Zamotajlov 1999). They can be readily differentiated from other Patrobini from China by having all tarsi pubescent dorsally. The other seven *Deltomerodes* species were recorded in Nepal and North India (Schmidt 1994, 1995, 1996, 1998; Schmidt and Hartmann1998; Zamotajlov 1999).

During sorting of the results of a recent expedition in Xizang, two series of specimens from Mêdog and Cona counties were readily recognized as two new species of the genus *Deltomerodes*. Therefore, the main purpose of this paper is to describe these two new species and provide an updated key to all known Chinese *Deltomerodes* species.

## Materials and methods

The specimens examined, including all types of the new species, are deposited in the collections of the Institute of Zoology, Chinese Academy of Science, Beijing, China (**IZAS**). The specimens were collected under stones or by pitfall traps and kept in zip-lock plastic bags with tissue paper soaked in 75% ethanol. The male genitalia were treated with a 10% KOH solution at room temperature for 12 h to show features of the endophallus. The female genitalia were treated with a 10% KOH solution at room temperature for 12 h, then stained in chlorazol black for 10 s and rinsed with 95% ethanol for 10 s. The male endophallus and the female genitalia were captured in a solution of glycerine. Images of the habitus and characters were captured with a Nikon D7500 camera attached to a Nikon SMZ18 microscope, then stacked with the software Helicon Focus 6.7.1 (Shi et al. 2013). Terminology for female genitalia follows Deuve (1993).

Abbreviations of measurements used in the paper are as follows: L, overall length from apex of mandibles to apex of elytra; HW, width of head, as greatest transverse distance of head; PL, length of pronotum, as linear distance from anterior to basal margin, measured along midline; PW, width of pronotum, as greatest transverse distance of pronotum; EL, length of elytra, as linear distance from anterior end of elytral lateral groove to apex of elytra, measured along elytral suture; EW, width of elytra, as greatest transverse distance of closed elytra. Abbreviations used for parts of the thorax are as follows: msem, mesepimeron; mset, mesepisternum; msst, mesosternum; mtet, metepisternum; mtst, metasternum. Abbreviations used for female genitalia are as follows: co, common oviduct; bc, bursa copulatrix; bcs, annular sclerite of bursa copulatrix; ep IX, epipleurite IX; gc I, gonocoxite I; gc II, gonocoxite II; sg, spermathecal gland; sd, spermathecal duct; sp, spermatheca.

## Taxonomy

### Deltomerodes

Taxon classificationAnimaliaColeopteraCarabidae

Deuve, 1992

53FE6CCB-1751-5A45-A30A-CC85AD453FA1

#### Type species.

*Deltomerodes memorabilis* Deuve, 1992.

#### Diagnosis.

The genus can be identified by the following combination of character states: Slender, medium-sized Partrobini, body elongate, length 8.5–12.0 mm; dorsal side black to reddish-brown. Head large with small eyes; first antennomere unisetose or with one seta distinctly longer than the others; two to four pairs of setae between eyes and neck constriction; mandibles normal, right mandible with a tooth beneath basal inner margin; terminal maxillary palpomere fusiform, broadest in middle; submentum with two pairs of setae in males, one to four pairs (usually two) in females. Pronotum ovate, longitudinal, cordate to subquadrate; lateral margins usually with one pair of setae before middle (two pairs in *D. memorabilis*); posterior angles usually with one pair of setae (two or three pairs in *D. schmidti*). Elytra oblong-ovate, shoulders rather narrow, oblique to slightly angulate; discal setiferous pores present on the third interval, usually also on the fifth and/or seventh intervals; striae more or less smoothed laterally. Thorax with suture between mesepisternum and mesosternum joining anterior margin of mesepimeron. All tarsomeres pubescent dorsally; fifth meso- and metatarsomeres with two to five pairs of setae ventrally. Apical lamella of aedeagus narrow, more or less bent dorsally in lateral view and twisted rightwards in dorsal view; endophallus with two groups of copulatory pieces: the proximal one near middle, usually loop-shaped (straight in *D. conaensis* sp. nov.); one or two spiniform distal pieces near distal margin of apical orifice. Parameres with short apical projections, left one somewhat obtuse or truncated at apex. Female gonopods dimerous, gonocoxite I glabrous; gonocoxite II thin, with one or two small subapical setae in one fovea; reproductive tract with a well-marked annular sclerotized ring on bursa copulatrix.

#### Comparision.

*Deltomerodes* is rather similar to *Deltomerus* and *Platidiolus*, and these are the only three genera with tarsi pubescent dorsally in Patrobini. *Deltomerus* also shares some other characters with *Deltomerodes*, including: body slender; fifth and seventh intervals of elytra usually with additional discal setiferous pores; fifth meso- and metatarsomeres with several setae ventrally. But *Deltomerus* is different in many characters: (1) first antennomere plurisetose, with several long setae; (2) submentum with more than two pairs of setae in males; (3) pronotum lateral margins plurisetose before middle (in *Deltomerodes*, only *D. memorabilis* with two pairs of mid-lateral setae); (4) all elytral striae distinct, subequally developed; (5) basal loop-shaped sclerite of endophallus absent (in *Deltomerodes*, only *D. conaensis* sp. nov. without basal loop-shaped sclerite). *Platidiolus* is differentiated from *Deltomerodes* by: (1) smaller size, body length less than 6 mm; (2) apical maxillary palpomere broadest at base, attenuate towards apex; (3) submentum with more than two pairs of setae in males; (4) elytra with discal setiferous pores only on the third interval.

### Checklist of *Deltomerodes* species

1 D. murzini Zamotajlov, 1999: 238: China (Yunnan: Yulongxueshan)
2 D. memorabilis Deuve, 1992: 82: China (Yunnan: Habaxueshan)
3 D. kryzhanovskii Zamotajlov, 1999: 244: China (Sichuan: Zhuotala)
4 D. miroshnikovi Zamotajlov, 1999: 242: China (Sichuan: Cholashan)
5 D. ovicollis sp. nov.: China (Xizang: Doxong La)
6 D. zolotichini zolotichini Zamotajlov, 1999: 250: China (Xizang: Serkyimla)
6’ D. zolotichini similis Zamotajlov, 1999: 251: China (Xizang: Serkyimla)
7 D. conaensis sp. nov.: China (Xizang: Rama La)
8 D. wrasei Zamotajlov, 1999: 240: China (Xizang: Gangdisêshan)
9 D. stenomus (Andrewes, 1936: 61): India (Sikkim: Jalep)
10 D. schawalleri schawalleri Schmidt, 1998: 6: Nepal (Province No. 1: Tangjala)
10’ D. schawalleri miniangularis Schmidt, 1998: 8: Nepal (Province No. 1: Meropapala)
11 D. sciakyi Schmidt, 1996: 145: Nepal (Province No. 1: Dingboche)
12 D. schmidti Zamotajlov, 1999: 248: Nepal (Province No. 1: Mt. Everest)
13 D. chulii Schmidt, 1995: 19: Nepal (Gandaki: Manaslu Himal)
14 D. nepalensis nepalensis Schmidt, 1994: 132: Nepal (Gandaki: Muktinath Himal)
14’ D. nepalensis gracilis Schmidt, 1995: 20: Nepal (Gandaki: Pisang Himal)
15 D. grilli Schmidt & Hartmann, 1998: 33: Nepal (Karnali: Sisne Himal)

### Key to Chinese species of *Deltomerodes*

**Table:** 

1	Fifth meso- and metatarsomeres with two pairs of setae ventrally (Fig. 5); body very slender, humeri narrow and oblique	**2**
–	Fifth meso- and metatarsomeres with three to five pairs of setae ventrally (Fig. 17); body less slender, humeri distinct, rounded to slightly angulate	**3**
2	Head with four pairs of setae between eyes and neck constriction; first antennomere unisetose; pronotum lateral margins with two pairs of setae before middle; sides of pronotum strongly sinuate before posterior angles, posterior margin slightly rounded, not protruded; seventh elytral interval with three setiferous pores; Yunnan (Habaxueshan)	***D. memorabilis***
–	Head with three pairs of setae between posterior margins of eyes and neck constriction; first antennomere plurisetose; pronotum lateral margins with one pair of setae before middle; sides of pronotum straight before posterior angles, posterior margin strongly protruded medially; seventh elytral interval with four to six setiferous pores; Xizang (Doxong La)	***D. ovicollis* sp. nov.**
3	Elytral fifth interval without setiferous pores; apical lamella of male genitalia broader, gradually acuminate at apex (Zamotajlov 1999: 243, figs 29–40); Xizang	**4**
–	Elytral fifth interval with one to seven setiferous pores; apical lamella of male genitalia narrower, abruptly acuminate at apex (male of *D. kryzhanovskii* not known); Sichuan, Yunnan	**7**
4	Pronotum cordate, strongly narrowed to base, its sides with distinct sinuation before posterior angles; Xizang (Serkyimla)	**5**
–	Pronotum subcordate or subquadrate, less narrowed to base, its sides with imperceptible or slight sinuation before posterior angles	**6**
5	Aedeagus larger, apex more markedly curved dorsally, apical copulatory piece longer, apex of parameres broader (Zamotajlov 1999: 245, figs 41–48)	***D. zolotichini zolotichini***
–	Aedeagus smaller, apex less markedly curved dorsally, apical copulatory piece shorter, apex of parameres narrower	***D. zolotichini similis***
6	Elytral scutellar stria less developed; sides of pronotum slightly sinuate before posterior angles, which are subrectangular, slightly protruding laterally; endophallus with two distal spiniform pieces, basal loop-shaped sclerite of endophallus absent; Xizang (Rama La)	***D. conaensis* sp. nov.**
–	Elytral scutellar stria developed; sides of pronotum not sinuate before posterior angles, which are obtuse, not protruding laterally; endophallus with one distal spiniform piece, basal loop-shaped sclerite of endophallus present; Xizang (Gangdisêshan)	***D. wrasei***
7	Head with three pairs of setae between eyes and neck constriction; both elytral scutellar pore and stria absent; female with four setae on each side of submentum; pronotum about as wide as head (PW/HW = 1.03–1.08); Yunnan (Yulongxueshan)	***D. murzini***
–	Head with two pairs of setae between eyes and neck constriction; both elytral scutellar pore and stria present; female with two setae on each side of submentum; pronotum slightly wider than head (PW/HW = 1.10–1.11); Sichuan	**8**
8	Pronotum anterior angles distinct, lateral margins with more or less distinct sinuation before hind angles; shoulders rounded; elytral striae shallow, with indistinct and irregular punctures, fifth interval with two to four setiferous pores, umbilicate series composed of ten to fourteen pores; Sichuan (Cholashan)	***D. miroshnikovi***
–	Pronotum anterior angles indistinct, lateral margins without sinuation before hind angles; shoulders angulate; elytral striae deep, with large and regular punctures, fifth interval with one or two setiferous pores, umbilicate series composed of nine or ten pores; Sichuan (Zhuotala)	***D. kryzhanovskii***

### Deltomerodes ovicollis

Taxon classificationAnimaliaColeopteraCarabidae

sp. nov.

1042D50E-3515-5E53-9718-5635F0C47BD5

http://zoobank.org/6ACFCC70-BE8C-415C-B279-B86E37B771BD

Figs 1 , 2–6 , 7–10 , 11–14

#### Type locality.

China, Xizang, Mêdog County, Doxong La (= Duoxiong La, 29.4890°N, 94.9527°E, 4210 m).

#### Type material.

***Holotype*:** male (IZAS), “Xizang, Mêdog County, Doxong La pass, 29.4860°N, 94.9527°E, 4210m, 2011 VII. 22, Yang XD lgt.”; “HOLOTYPE♂ *Deltomerodes ovicollis* sp. nov., des. YAN & SHI, 2021” [red label]. ***Paratypes***: 1 female (IZAS), same data as holotype but labeled as paratype; 4 females (IZAS), “China, Tibet, Lage to Pai Township. foot path. pitfall trap. 29.4897°N, 94.9533°E”; “4090 m. 2015. 8.8-10. D3. Liang H. B. coll. IOZCAS”.

#### Diagnosis.

The new species is unique in the genus *Deltomerodes* in having the following two features: (1) pronotum ovate with posterior margin strongly protruded medially; (2) first antennomere with two or three short accessory setae. In addition, the new species can be diagnosed from congeners by the combination of following characters: head with three pairs of setae between eyes and neck constriction; pronotum lateral margins with one pair of setae before middle, not sinuate before posterior angles; elytral discal pores present on third, fifth and seventh intervals; fifth meso- and metatarsomeres with two pairs of setae ventrally; armature of endophallus consisting of a large loop-shaped proximal copulatory piece and a long spiniform distal piece.

#### Description.

**Figure 1.**
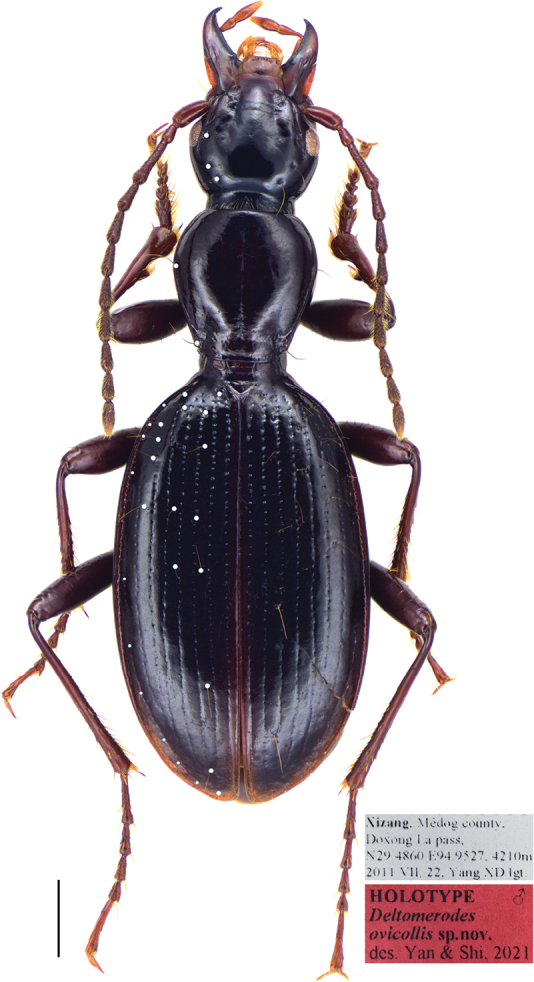
*Deltomerodes ovicollis* sp. nov. (holotype). Habitus and labels (the white dots represent the setiferous pores on the left side of the specimen). Scale bar: 1.0 mm.

Habitus as in Figure 1. Medium-sized for a Patrobini species (L = 9.3–10.7 mm; EW = 3.3–3.5 mm).

***General appearance*:** Dorsal side dark brown to reddish-brown, shiny, without metallic luster; head, pronotum and elytra brown to reddish-brown; mandibles, antennae and legs a little paler, palpi reddish-brown; ventral side largely dark brown to reddish-brown. Dorsal surface of forebody smooth except for basal foveae of pronotum slightly punctate. Microsculpture invisible on head and pronotum, isodiametric on the entire elytra.

**Figures 2–6.**
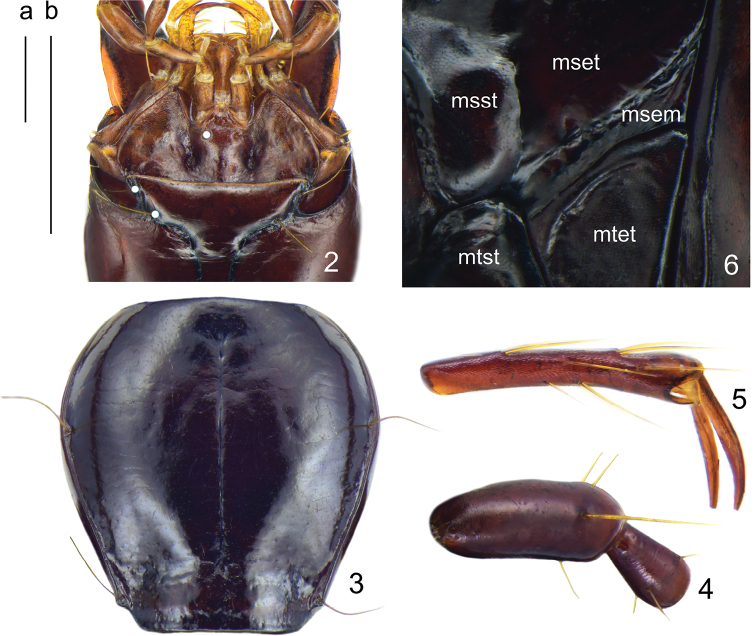
*Deltomerodes ovicollis* sp. nov. (holotype) **2** labium **3** pronotum **4** first two segments of antenna **5** lateral side of the fifth metatarsomere **6** ventral side of thorax. Scale bars: 0.5 mm (a for **2, 3**, b for **4–6**).

***Head*:** Broad and ovate; surface smooth, without distinct punctures. Mandibles not elongated, right mandible with a weakly protruding tooth beneath the basal inner margin; terminal maxillary palpomere broadest in the middle, penultimate and antepenultimate palpomeres with an apical ring of setae; ligula with two adjacent apical setae. Antennae pubescent beginning with third antennomere; first antennomere plurisetose, with two or three short setae and one primary seta distinctly longer than the others; second antennomere glabrous except for apical ring of four to five setae (Fig. 4). Eyes small, very slightly convex; temporae tumid, much longer than eye diameter; neck constriction rather deep. Frontal furrows short, shallow but distinct, extended to level of middle of eyes, slightly divergent posteriorly, finely rugose anteriorly. Head with three setae between eyes and neck constriction on each side: one supraorbital seta near middle level of eyes (in one female with one additional seta on one side), two setae between posterior margins of eyes and neck constriction, a little distant from the neck constriction. Mentum with two deep longitudinal foveae basally, tooth narrow and bifid, apical notch shallow; submentum with two setae on each side in both sexes (Fig. 2).

***Pronotum*:** Longitudinal ovate, rather narrow, PW/PL = 0.95–1.05, slightly wider than head, PW/HW = 1.12–1.25, widest near anterior third, fairly convex, moderately constricted posteriorly. Anterior margin near straight; anterior angles indistinct, not protruding forward; lateral margins broadly rounded in front, without sinuation before posterior angles; posterior margin strongly protruded, forming a wide median peduncle and short incisions near posterior angles (Fig. 3); posterior angles obtuse, their apices slightly pointed outwards. Anterior transverse impression shallow and smooth; basal foveae shallow, finely punctate and wrinkled; disc smooth, median line shallow but distinct, obliterated at both extremities; lateral grooves extremely narrow, not punctate. Lateral margins each with one seta near anterior third, another seta situated a little before posterior angle.

***Elytra*:** Oblong-ovate, convex; EL/EW = 1.62–1.74, EW/PW = 1.70–1.83, EL/PL = 2.75–2.95, widest near posterior third, constricted both anteriorly and posteriorly; humeri narrow and oblique, humeral tooth hardly visible; lateral groove narrow. Intervals slightly convex; inner striae well incised, coarsely punctate basally, lateral striae shallower and finer punctate; scutellar stria inconspicuous, only with one or two punctures and a superficial impression, scutellar pore present at base of second interval or second stria; third interval with five to seven setiferous pores, adjoining the third stria or placed on the third interval, the first one near base; fifth interval with three to seven setiferous pores, all in basal half of elytra; seventh interval with four to six setiferous pores in basal half; umbilicate series composed of ten to thirteen pores, basal ones not aggregated.

***Ventral side*:** Largely smooth, lateral areas of mesepisternum very finely wrinkled; mesepimeron narrow, slightly widened laterally, suture separating mesepisternum and mesosternum joins anterior margin of mesepimeron (Fig. 6); metepisternum long and narrow, not punctate. Lateral areas of abdominal sternites finely rugose, sternites IV to VI each with one pair of setae near middle; sternite VII with one seta on each side in males and two in females. Female sternite VIII with anterolateral apophyses long, longitudinal depigmentation distinct, posterointernal depigmentation present (Fig. 13). Female tergite VIII with anterolateral apophyses quite short; longitudinal carinae absent, transverse ones short and laterally placed, median sclerotization absent, basal depigmentation present, apical depigmentation absent (Fig. 12).

***Legs*:** Males with the basal two protarsomeres slightly expanded, second protarsomere distinctly wider than third, which is near triangular; fourth protarsomere slightly bilobed; metatrochanter normal, apex not protruding; all tarsomeres pubescent dorsally; fifth meso- and metatarsomeres with two pairs of setae ventrally (Fig. 5).

**Figures 7–10.**
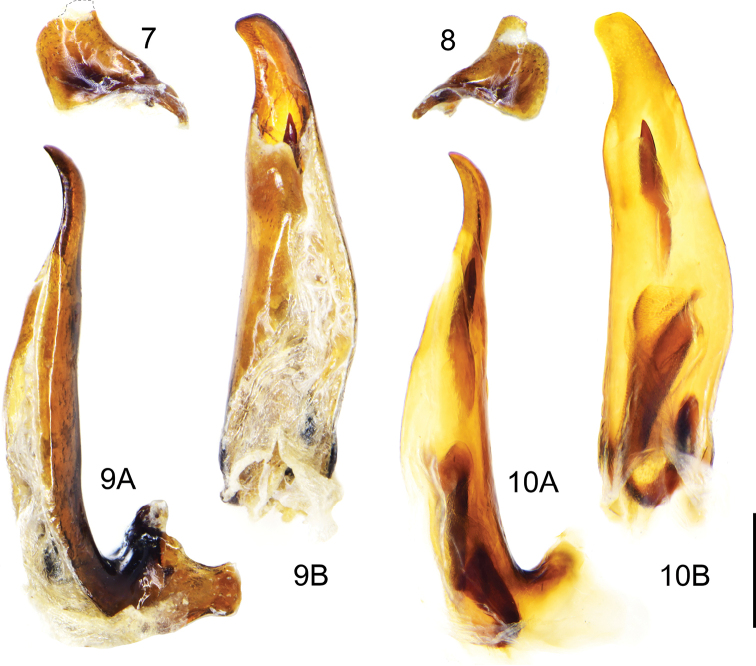
*Deltomerodes ovicollis* sp. nov. (holotype) **7** left paramere of aedeagus (dotted line shows damaged apex) **8** right paramere of aedeagus **9** median lobe of aedeagus, left lateral view (**9A**), dorsal view (**9B**) **10** endophallus of aedeagus, left lateral view (**10A**), dorsal view (**10B**). Scale bar: 0.5 mm.

***Male genitalia*:** Median lobe of aedeagus strongly bent at base, gutter-shaped and opened dorsally; in lateral view apical lamella slightly curved dorsally; in dorsal view apical lamella gradually attenuated towards apex and fairly twisted rightwards, apex round-truncate, forming a faint hook to the right (Fig. 9). Armature of endophallus consisting of a large loop-shaped proximal copulatory piece and a long spiniform distal piece (Fig. 10). Left paramere larger than right one, both short and gradually contracted towards apex but left one more truncated at apex, both with one long apical seta and one or two short subapical setae (Figs 7, 8).

**Figures 11–14.**
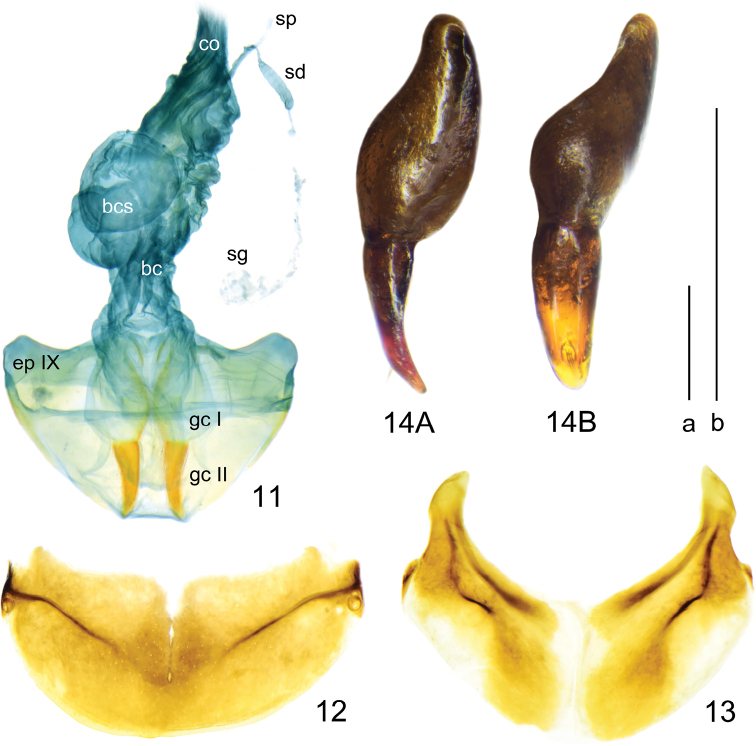
*Deltomerodes ovicollis* sp. nov. (paratype female) **11** female reproductive tract **12** female tergite VIII **13** female sternite VIII **14** gonocoxa, right lateral view (**14A**), dorsal view (**14B**). Scale bars: 0.5 mm (a for **11–13**, b for **14**).

***Female genitalia*:** Gonocoxite I glabrous; gonocoxite II narrow and slightly curved, blunt-rounded at apex, with two small setae in one fovea at subapical inner margin (Fig. 14). Reproductive tract with well-marked annular sclerotized ring about 0.37 mm in diameter on bursa copulatrix, spermatheca small and tubular, spermathecal duct well developed, spermathecal gland inserted near apex of spermathecal duct, much longer than spermathecal duct (Fig. 11).

#### Distribution.

**Map 2.**
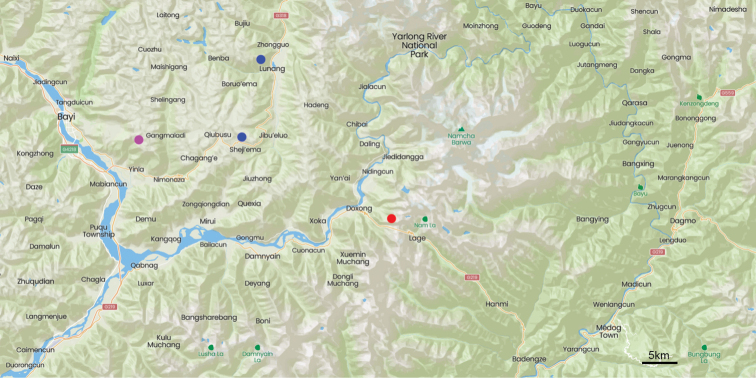
Distribution map for *Deltomerodes ovicollis* sp. nov. and adjacent taxa: *D. ovicollis* sp. nov. (red); *D. zolotichini zolotichini* (blue); *D. zolotichini similis* (magenta).

This species is known only from the type locality of Doxong La in Mêdog County, Xizang (Map 2).

#### Etymology.

The scientific name of the new species comes from Latin and refers to its very special ovate pronotum.

#### Remarks.

Zamotajlov (1999) recognized five species groups in the genus *Deltomerodes*, among which three were from China. Most of the Chinese species (four of six) were assigned to the *murzini*-group, whereas *D. zolotichini* and *D. memorabilis* are in their own species groups. According to Zamotajlov (1999), the new species can be keyed to the *murzini*-group for some characters: (1) head with less than four pairs of setae between eyes and neck constriction; (2) pronotum not cordate, without distinct sinuation before posterior angles, sides with one seta in anterior third. However, it is quite different from all other members of this species group not only for its very special pronotum shape (ovate with posterior margin strongly protruded) but also for other important features: (1) fifth meso- and metatarsomeres with two pairs of setae ventrally vs. with three or four pairs of setae in other species of this group; (2) first antennomere with two or three short accessory setae vs. without short setae; (3) seventh elytral interval with four or more discal pores vs. with one or two setiferous pores. Additionally, the new species is more similar to *D. memorabilis* mainly in aspects of the humeral shape and in the chaetotaxy of the elytral intervals and fifth tarsomeres, though the differences between the two species are rather distinct following the key. Thus, the new species may be either assigned to the *memorabilis*-group or assigned to its own group.

### Deltomerodes conaensis

Taxon classificationAnimaliaColeopteraCarabidae

sp. nov.

886987A3-A2E9-5F5D-A34A-F26F13793CE9

http://zoobank.org/52BCBE25-A05D-4FC4-A151-C81D2380673A

Figs 15 , 16–19 , 20–23 , 24–28

#### Type locality.

China, Xizang: Cona County, Rama La pass (28.3037°N, 91.8834°E, 5019 m).

#### Type material.

***Holotype*:** male (IZAS), “Xizang, Lhoka pref., Cona county, Quchomo Xiang, Rama La pass, alpine meadow, 28.3037°N, 91.8834°E, 5019m”; “ under stone, 2019. VII. 24, Shi HL, Yan WF & Zhu PZ lgt., Exp. BJFU 2019”; “HOLOTYPE♂ *Deltomerodes conaensis* sp. nov., des. YAN & SHI, 2021” [red label]. ***Paratypes***: 9 males and 7 females (IZAS), same data as holotype but labeled as paratypes.

#### Diagnosis.

The new species can be distinguished within the genus by the combination of: head generally with two pairs of setae between eyes and neck constriction; pronotum subcordate, lateral margins slightly sinuate before posterior angles, which are slightly pointed outwards; humeri distinct, rounded; scutellar pore present; elytral discal pores only present on third and seventh intervals; fifth meso- and metatarsomeres with three to four pairs of setae ventrally; endophallus consisting of a straight and weakly sclerotized proximal copulatory piece and two distal spiniform pieces.

#### Description.

**Figure 15.**
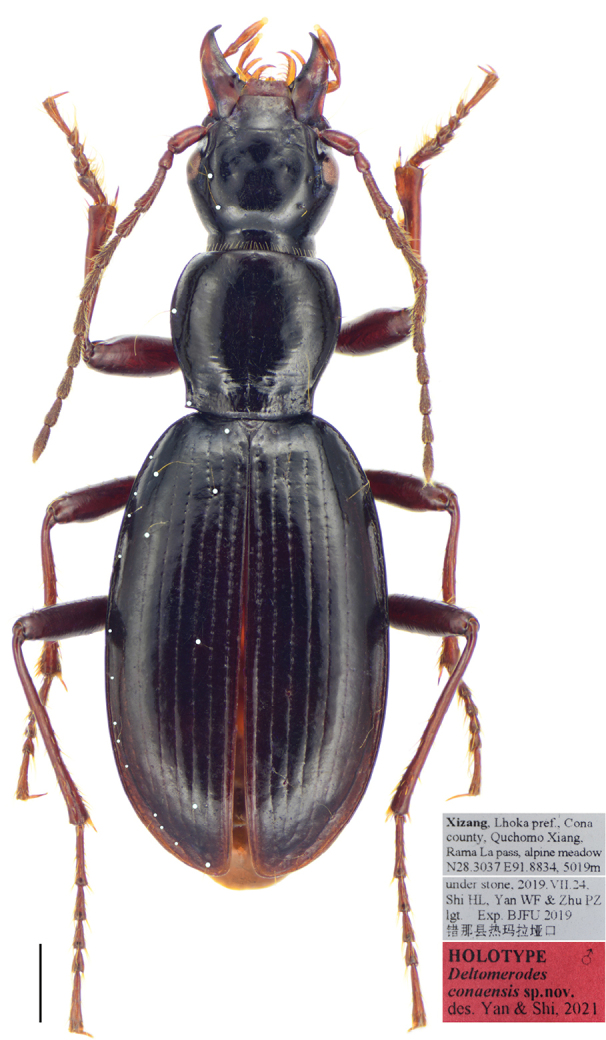
*Deltomerodes conaensis* sp. nov. (holotype). Habitus and labels (the white dots represent the setiferous pores on the left side of the specimen). Scale bar: 1.0 mm.

Habitus as in Figure 15. Medium-sized for a Patrobini species (L = 9.7–11.5 mm; EW = 3.2–3.7 mm).

***General appearance*:** Dorsal side dark brown to black, shiny, without metallic luster; head, mandibles, pronotum and elytra brown; antennae and legs a little paler, palpi reddish-brown; ventral side largely dark brown, abdominal sternum brown. Dorsal side smooth except for basal foveae of pronotum slightly punctate. Microsculpture invisible on head and pronotum, isodiametric on elytra, distinct in apical third, hardly visible near base.

**Figures 16–19.**
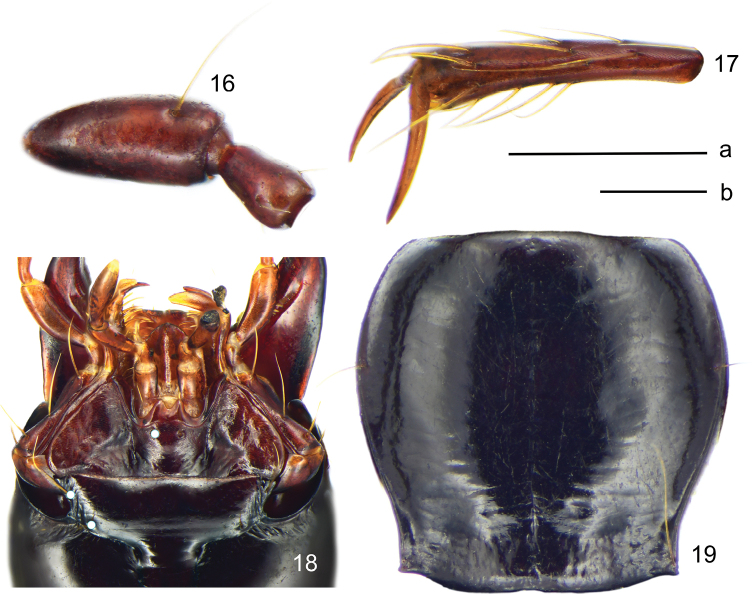
*Deltomerodes conaensis* sp. nov. (holotype) **16** first two segments of antenna **17** lateral side of fifth metatarsomere **18** labium **19** pronotum. Scale bars: 0.5 mm (a for **16, 17**, b for **18, 19**).

***Head*:** Broad and ovate; surface smooth, without coarse punctures. Mandibles not elongated, right mandible with a weakly protruding tooth beneath basal inner margin; terminal maxillary palpomere broadest in middle; ligula with two adjacent apical setae. Antennae pubescent beginning with third antennomere; first antennomere with only one long seta; second antennomere glabrous except for apical ring of setae (Fig. 16). Eyes small, very slightly convex; temporae tumid, much longer than eye diameter; neck constriction shallower than in *D. ovicollis* sp. nov. Frontal furrows short and rather shallow, extended to level of posterior edge of eyes, slightly divergent posteriorly; with two setae between eyes and neck constriction on each side (two specimens with three or four setae on one side), including one supraorbital seta near middle level of eyes, and one seta between posterior margins of eyes and neck constriction, not adjoining to neck constriction. Mentum with two deep longitudinal foveae basally, tooth narrow and bifid, apical notch shallow; submentum with two setae on each side in both sexes (Fig. 18).

***Pronotum*:** Longitudinal-subcordate, rather narrow, PW/PL = 1.00–1.15, slightly wider than head, PW/HW = 1.11–1.21, widest near anterior third, fairly convex, moderately constricted posteriorly. Anterior margin near straight; anterior angles rounded, somewhat distinct, not protruding anteriorly; lateral margins broadly rounded in front, with slight sinuation before posterior angles; posterior margin nearly straight to slightly rounded; posterior angles obtuse to rectangular, apex sharp, slightly pointed outwards. Anterior transverse impression shallow and smooth; basal foveae shallow, finely punctate and wrinkled; disc smooth, median line shallow, obliterated at both extremities; lateral grooves narrow, not punctate. Lateral margins each with one seta before middle (two specimens with two setae on one side or both sides), another seta situated a little before posterior angle (Fig. 19).

***Elytra*:** Oblong-ovate, convex; EL/EW = 1.69–1.74, EW/PW = 1.64–1.74, EL/PL = 2.80–2.84, widest near posterior third; humeri distinct, rounded, with small humeral tooth. Intervals slightly convex; inner striae well incised, finely punctate basally, lateral striae shallower; scutellar stria inconspicuous, only with one or two punctures and a superficial impression, scutellar pore present at base of second interval; third interval with three to four setiferous pores, all adjoining the third stria, the first one near basal sixth; seventh interval with one or two setiferous pores near basal sixth; umbilicate series composed of nine to twelve pores, basal ones not aggregated.

***Ventral side*:** Prosternum smooth, propleuron densely punctate; mesosternum and mesopleuron wrinkled, mesopleuron with sporadic coarse punctures; mesepimeron narrow, slightly widened laterally, suture separating mesepisternum and mesosternum joins anterior margin of mesepimeron; metepisternum rather long and narrow, not punctate. Lateral areas of abdominal sternites finely rugose, sternites IV to VI each with one pair of setae near middle; sternite VII with one seta on each side in males and two in females. Female sternite VIII with anterolateral apophyses long, with both longitudinal and transverse depigmentation distinct, posterointernal depigmentation present (Fig. 26). Female tergite VIII with anterolateral apophyses quite short; longitudinal carinae absent, transverse ones short and laterally placed, median sclerotization absent, basal depigmentation indistinct, apical depigmentation absent (Fig. 25).

***Legs*:** Males with basal two protarsomeres slightly expanded, second protarsomere distinctly wider than third, which is near triangular; fourth protarsomere slightly bilobed; metatrochanter normal, apex not protruding; all tarsomeres pubescent dorsally; fifth meso- and metatarsomeres with three or four pairs of setae ventrally (Fig. 17).

**Figures 20–23.**
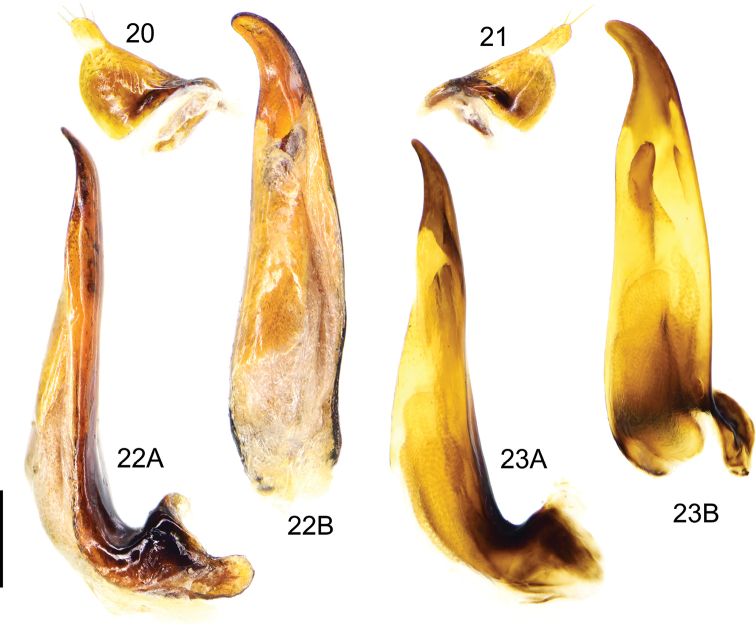
*Deltomerodes conaensis* sp. nov. (holotype) **20** left paramere of aedeagus **21** right paramere of aedeagus **22** median lobe of aedeagus, left lateral view (**22A**), dorsal view (**22B**) **23** endophallus of aedeagus, left lateral view (**23A**), dorsal view (**23B**). Scale bar: 0.5 mm.

***Male genitalia*:** Median lobe of aedeagus strongly bent at base, gutter-shaped and opened dorsally; in lateral view apical lamella moderately curved dorsally; in dorsal view apical lamella gradually attenuated towards apex and faintly twisted rightwards, apex rounded, forming a prominent hook to the right (Fig. 22). Armature of endophallus consisting of a straight and weakly sclerotized proximal copulatory piece and two spiniform distal pieces, the dorsal one large and stout, the left one much narrower (Fig. 23). Left paramere larger than right one, both short and gradually contracted towards apex but the left one more truncated at apex, both with two long apical setae and one or two short subapical setae (Figs 20, 21).

**Figures 24–28.**
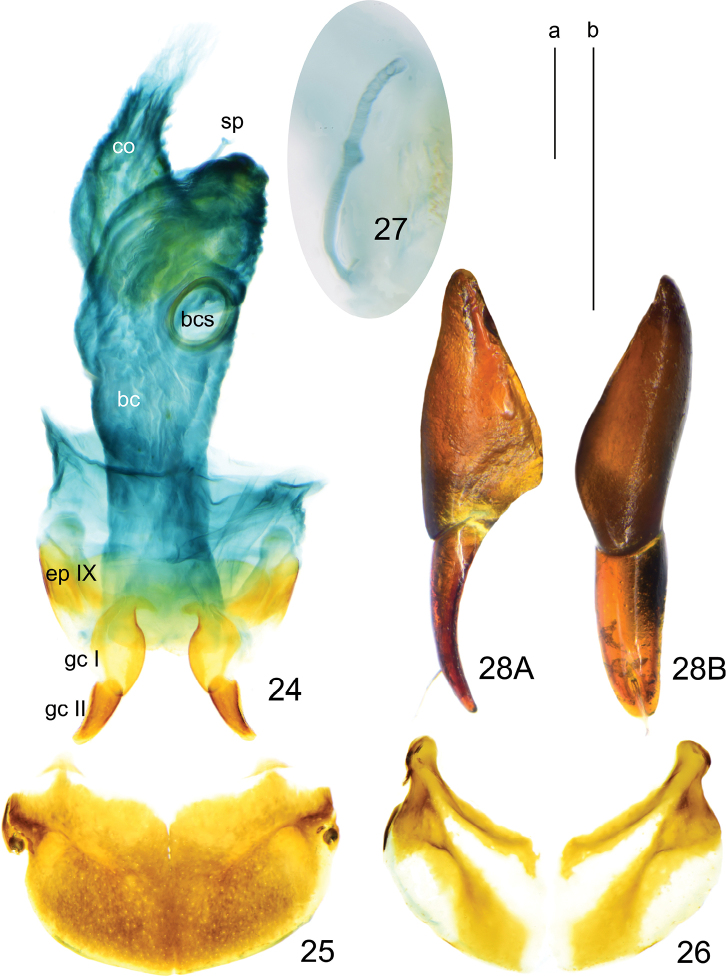
*Deltomerodes conaensis* sp. nov. (paratype female) **24** female reproductive tract **25** female tergite VIII **26** female sternite VIII **27** spermatheca **28** gonocoxa, right lateral view (**28A**), dorsal view (**28B**). Scale bars: 0.5 mm (a for **24–26**, b for **27, 28**).

***Female genitalia*:** Gonocoxite I glabrous; gonocoxite II narrow and slightly curved, blunt-rounded at apex, with two small setae in one fovea at subapical inner margin (Fig. 28). Reproductive tract with well-marked annular sclerotized ring about 0.35 mm in diameter on bursa copulatrix; spermatheca tubular, very small, without spermathecal duct and spermathecal gland (Figs 24, 27).

#### Distribution.

**Map 3.**
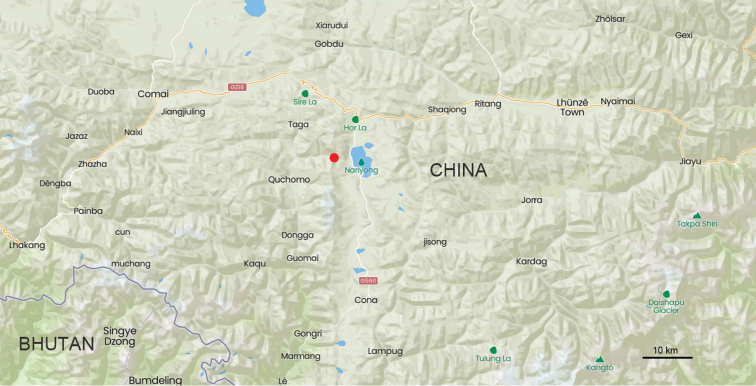
Distribution map for *Deltomerodes conaensis* sp. nov. (red).

This species is known only from the type locality of Rama La in Cona County, Xizang (Map 3).

#### Etymology.

The scientific name of the new species refers to its type locality in Cona County.

#### Remarks.

According to the key provided by Zamotajlov (1999), the new species belongs to the *murzini*-group for some characters: (1) head with less than four pairs of setae between eyes and neck constriction; (2) pronotum not cordate, without distinct sinuation before posterior angles, sides with one seta in anterior third; (3) fifth meso- and metatarsomeres with three or four pairs of setae ventrally. Within this species group, the new species seems to be closest to *D. wrasei* for their adjacent distribution and morphological similarities in chaetotaxy, pronotum shape and in the apical lamella of the aedeagus. However, the armature of the endophallus in *D. conaensis* sp. nov. has a straight and weakly sclerotized proximal copulatory piece. The new species is different from all other known species of the genus, in which the proximal copulatory piece is well-sclerotized and loop-like.

## Discussion

The genus *Deltomerodes* is well defined by its particular appearance and morphological characters, while relationships among its members remain unclear. Therefore, more materials are needed for a more extensive phylogenetic analysis, especially in consideration of some undetermined species with only females available and an insufficient number of specimens from the potential distribution area of the genus, including Southern Xizang and Bhutan.

The specimens of *D. conaensis* sp. nov. were found under stones along a dry creek bed formed by seasonal snow melting in the alpine meadow macrohabitat at about 5000 m altitude. The new species of *Deltomerodes*co-occur with *Nebria superna* Andrewes, a supra-high-altitude species widely distributed in the Tibetan Plateau. It is noticeable that all specimens of *D. conaensis* sp. nov. were found only in a small (about twenty square meters) area slightly elevated above the creek bed slightly protruding from the creek bed, while *Nebria superna* was found extensively all along the creek bed. This suggests that species of *Deltomerodes* are more stenoecic and occur only in some rather humid microhabitats at high altitudes.

## Supplementary Material

XML Treatment for Deltomerodes

XML Treatment for Deltomerodes ovicollis

XML Treatment for Deltomerodes conaensis

## References

[B1] Andrewes HE (1936) Papers on Oriental Carabidae – XXX. Annals and Magazine of Natural History (10) 18: 54–65. 10.1080/00222933608655174

[B2] Deuve T (1992) *Deltomerodes memorabilis* n. gen., n. sp., carabique Patrobinae des hautes montagnes du Yunnan. Revue Française d’Entomologie (N.S.) 14: e82.

[B3] Deuve T (1993) L’abdomen et les genitalia des femelles de Coléoptères Adephaga. Mémoires du Muséum d’Histoire Naturelle. Zoologie 155: e184.

[B4] Kirby W (1837) Part the fourth and last. The insects. In: Richardson J (Ed.) Fauna Boreali-Americana; or the zoology of the northern parts of British America: containing descriptions of the objects of natural history collected on the late Northern Land Expeditions, under command of captain Sir John Franklin, R.N. Norwich: J. Fletcher, [xxxix +] 329 [+ 1] pp.[, 8 pls].

[B5] Schmidt J (1994) Beschreibungen neuer Arten der Carabidae aus Nepal (1): Gattungen *Trechus*, *Deltomerodes*, *Pterostichus* (Insecta: Coleoptera). Reichenbachia 30(21): 129–135.

[B6] Schmidt J (1995) Beschreibungen neuer Arten der Carabidae aus Nepal (2): Gattungen *Deltomerodes*, *Pterostichus*, *Xestagonum* (Insecta: Coleoptera). Reichenbachia 31(5): 19–25.

[B7] Schmidt J (1996) Beschreibungen neuer Arten der Carabidae aus Nepal (3): Gattungen *Broscus*, *Deltomerodes*, *Xestagonum* (Insecta: Coleoptera). Reichenbachia 31(26): 143–154.

[B8] Schmidt J (1998) Beschreibungen neuer Arten der Carabidae aus Nepal (4): Gattungen *Broscus*, *Deltomerodes*, *Xestagonum* (Insecta: Coleoptera). Entomologische Abhandlungen und Berichte aus dem Staatlich Museum für Tierkunde in Dresden 58(1): 5–28.

[B9] Schmidt J Hartmann M (1998) *Deltomerodes grilli* sp. n., der erste Nachweis einer alpinen Art der Tribus Patrobini aus West-Nepal (Coleoptera, Carabidae). Mitteilungen der Münchner Entomologischen Gesellschaft 88: 33–36.

[B10] Shi HL Zhou HZ Liang HB (2013) Taxonomic synopsis of the subtribe Physoderina (Coleoptera, Carabidae, Lebiini), with species revisions of eight genera. ZooKeys 284: 1–129. 10.3897/zookeys.284.3983 PMC3677377 23794843

[B11] Zamotajlov AS (1999) Redefinition of the genus *Deltomerodes* Deuve, 1992, with the description of new species (ColeopteraCarabidaePatrobinae). In: Zamotajlov A, Sciaky R (Eds) Advances in Carabidology. Papers Dedicated to the Memory of Prof. Dr. Oleg L. Kryzhanovskij. Krasnodar: MUISO Publishers, 473 pp. [Pp. 229–258.]

[B12] Zamotajlov AS (2002) Inferring phylogenetic system of the carabid subfamily Patrobinae (Coleoptera, Carabidae). St. Petersburg: Zoological Institute of Russian Academy of Sciences, 145 pp. [Meetings in memory of N.A. Cholodkovsky; Iss. 55] [In Russian]

[B13] Zamotajlov AS (2017) Tribe Patrobini Kirby, 1837. Catalogue of Palaearctic Coleoptera, Volume 1: Archostemata – Myxophaga – Adephaga, 1477 pp. [p. 11, 456–465.]

